# Metabolomics guided pathway analysis reveals link between cancer metastasis, cholesterol sulfate, and phospholipids

**DOI:** 10.1186/s40170-017-0171-2

**Published:** 2017-10-31

**Authors:** Caroline H. Johnson, Antonio F. Santidrian, Sarah E. LeBoeuf, Michael E. Kurczy, Nicholas J. W. Rattray, Zahra Rattray, Benedikt Warth, Melissa Ritland, Linh T. Hoang, Celine Loriot, Jason Higa, James E. Hansen, Brunhilde H. Felding, Gary Siuzdak

**Affiliations:** 1 Scripps Center for Metabolomics, The Scripps Research Institute, La Jolla, CA USA; 2 Department of Environmental Health Sciences, Yale School of Public HealthYale School of Medicine, New Haven, CT USA; 3 Yale Cancer Center, Yale School of Medicine, New Haven, CT USA; 4 Department of Molecular and Experimental Medicine, The Scripps Research Institute, La Jolla, CA USA; 5 Current address: Department of Molecular Oncology and Immunotherapies, StemImmune, Inc., San Diego, CA 92122 USA; 6 Current address: NYU Langone Medical Center, New York, NY 10016 USA; 7 Current address: CVMD IMED AstraZeneca, Gothenburg, Sweden; 8 Department of Therapeutic Radiology, Yale School of Medicine, Yale University, New Haven, CT 06520 USA; 9 Current address: Active Motif Inc, Carlsbad, CA 92008 USA

**Keywords:** Cancer, Cholesterol sulfate, Phospholipids, Autonomous metabolomics, Mummichog, Metastasis, XCMS

## Abstract

**Background:**

Cancer cells that enter the metastatic cascade require traits that allow them to survive within the circulation and colonize distant organ sites. As disseminating cancer cells adapt to their changing microenvironments, they also modify their metabolism and metabolite production.

**Methods:**

A mouse xenograft model of spontaneous tumor metastasis was used to determine the metabolic rewiring that occurs between primary cancers and their metastases. An “autonomous” mass spectrometry-based untargeted metabolomic workflow with integrative metabolic pathway analysis revealed a number of differentially regulated metabolites in primary mammary fat pad (MFP) tumors compared to microdissected paired lung metastases. The study was further extended to analyze metabolites in paired normal tissues which determined the potential influence of metabolites from the microenvironment.

**Results:**

Metabolomic analysis revealed that multiple metabolites were increased in metastases, including cholesterol sulfate and phospholipids (phosphatidylglycerols and phosphatidylethanolamine). Metabolite analysis of normal lung tissue in the mouse model also revealed increased levels of these metabolites compared to tissues from normal MFP and primary MFP tumors, indicating potential extracellular uptake by cancer cells in lung metastases. These results indicate a potential functional importance of cholesterol sulfate and phospholipids in propagating metastasis. In addition, metabolites involved in DNA/RNA synthesis and the TCA cycle were decreased in lung metastases compared to primary MFP tumors.

**Conclusions:**

Using an integrated metabolomic workflow, this study identified a link between cholesterol sulfate and phospholipids, metabolic characteristics of the metastatic niche, and the capacity of tumor cells to colonize distant sites.

**Electronic supplementary material:**

The online version of this article (10.1186/s40170-017-0171-2) contains supplementary material, which is available to authorized users.

## Background

The biology of metastatic cancer is complex, and cells that enter the metastatic cascade encounter a multitude of challenges. The fraction of malignant cells that are shed from the primary tumor require traits that allow them to survive in circulation and colonize distant organs [1]. Once seeded, the cells must adapt to the new pool of extracellular metabolites available and survive immune surveillance. To do this, cancer cells reprogram their microenvironment to assist tumor growth. Thus, the metabolism, growth, and survival of the metastatic cells are likely dependent on the attributes of the new site.

As cancer cells proliferate, they require energy to synthesize macromolecules, support cell growth, and maintain redox homeostasis [2, 3]. Sources of energy can be controlled by the expression of metabolic enzymes such as hexokinase which converts glucose to glucose-6-phosphate in the glycolysis pathway, as seen in many cancers, including glioblastoma multiforme [4]. However, cancer cells can also utilize transporters to help produce and import metabolites from the microenvironment. A recent study revealed that metastatic cells release an enzyme into the extracellular space catalyzing the phosphorylation of creatine. Phosphocreatine is then imported into disseminated cancer cells to generate adenosine triphosphate (ATP), fueling metastatic survival [5]. Moreover, stromal-epithelial metabolic coupling has been described for symbiotic nutrient sharing in cancer. A recent report revealed that adipocytes in the omentum provide fatty acids for primary ovarian cancers promoting rapid tumor growth and metastasis [6]. Metabolites can also be directly involved in increasing cancer cell growth, for example, succinate and fumarate are known to inhibit prolyl hydroxylase enzymes producing a pseudo-hypoxic state, driving glycolysis, and tumor proliferation [7]. Therefore, understanding the metabolism of cancer cells in a primary tumor versus those that have metastasized to a secondary organ site can reveal metabolic adaptions of a disseminating cancer cell.

A recently developed autonomous metabolomic workflow was implemented with integrative metabolic pathway analysis to identify metabolites and guide further biological investigation [8]. A MDA-MB-435 xenograft model was used to generate mammary fat pad (MFP) tumors in the mouse and spontaneous metastases to the lung, to test the hypothesis that metastatic cells undergo a metabolic adaption to survive the new microenvironment [9]. These cancer tissues were subjected to comprehensive metabolomics and pathway analyses, which revealed a number of metabolic dependencies for cancer cells that resided in these tissues.

## Methods

The aims of this study were to assess the metabolic differences between primary tumors and spontaneous metastases. To carry out a comprehensive analysis of metabolites in tissues, we used an autonomous mass spectrometry-based metabolomic workflow on primary tumor and metastasis tissue extracts [8].

### Cell culture

MDA-MB-435 cells were grown in EMEM supplemented with nonessential amino acids, vitamins, 2 mM l-glutamine, 1 mM pyruvate, and 10% fetal bovine serum.

### Animal treatment and sample collection

Six- to eight-week-old female C.B-17/SCID mice were injected with *F-luc*-tagged cancer cells: 2.5 × 10^5^ (30 μl) MDA-MB-435 cells into the fourth mammary fat pad. Primary tumors were removed surgically when they reached 300 mm^3^ in size (approximately 4 weeks post-injection), the endpoint. Mice were then examined weekly (IVIS 200, Xenogen) by non-invasive bioluminescence imaging, 10 min after i.p. injection of d-luciferin (100 mg/kg) to assess the presence of lung metastasis. Animals were sacrificed 24 h after bioluminescence signal on the chest reached 1 × 10^7^ photons/s/cm^2^. In order to eliminate any artefacts that could occur with the enzymatic procedure, i.p. injection of d-luciferin was not used for the final dissection. Animal work complied with the National Institutes of Health and institutional guidelines (TSRI is AAALAC accredited).

### Tissue sample extraction

Three sections were taken from different regions of mammary fat pad tumors (core, middle, edges) with a combined approximate weight of 15 mg. The tissues were added to high recovery glass vials (Agilent Technologies, Santa Clara, CA) on dry ice. Lung metastases were first assessed by bioluminescence imaging, as described above, to guide the microdissection under the microscope. The microdissected metastasis was then pooled for each mouse, then similarly placed in vials on dry ice. Each sample was homogenized in 400 μl methanol/water (4:1) and 1 mm glass beads (BioSpec Products, Bartlesville, OK) for 30 s at 3500 rpm. The homogenate was added to glass vials, sonicated for 10 min at room temperature, and stored at − 20 °C overnight. Samples were then centrifuged at 13,000 rpm for 15 min and the supernatant transferred to a new 1.5-ml centrifuge tube. The pellet was resuspended in 600 μl ice cold acetone, vortexed for 10 s, and sonicated for 10 min at room temp. The pellet samples were stored at − 20 °C for 1 h followed by centrifugation (13,000 rpm for 15 min). The supernatant was pooled with the supernatant collected earlier and dried down in a Speedvac for 4 h. The pellet was used for protein quantification by microBCA (Thermo Fisher Scientific, Waltham, MA).

Samples were resuspended in acetonitrile/water/isopropanol (50/40/10 *v*/*v*) according to the protein concentration; the lowest concentration was resuspended in 40 μl and the other samples adjusted accordingly. Each sample was then sonicated for 10 min, centrifuged at 13,000 rpm for 15 min, and the supernatants transferred to glass HPLC vials for LC/MS analysis.

### Untargeted metabolomic analysis

Samples were randomized and analyzed by high-performance liquid chromatography-electrospray ionization quadrupole time-of-flight mass spectrometry (HPLC-ESI-QTOFMS). A pooled sample containing 10 μl of each individual sample was injected (5 μl) every five injections for quality control. In addition, a water-based wash and blank were run after each sample. The samples were analyzed by hydrophilic interaction liquid chromatography (HILIC) analysis as previously described [10] using an Agilent 6550 ion funnel QTOF in ESI negative mode. HILIC analysis was used as we were aiming to identify the polar metabolites which have been previously shown to have roles in cancer metabolism [11]. Each sample (5 μl) was injected onto a Luna Aminopropyl column, 3 μm, 150 mm × 1.0 mm I.D. (Phenomenex), the mobile phase was composed of A = 20 mM ammonium acetate and 40 mM ammonium hydroxide in 95% water and B = 95% acetonitrile. The linear gradient elution from 100% B (0–5 min) to 100% A over 50 mins, held at 100% A for 5 min, and a 10-min post-run at a flow rate of 50 μl/min.

**Fig. 1:**
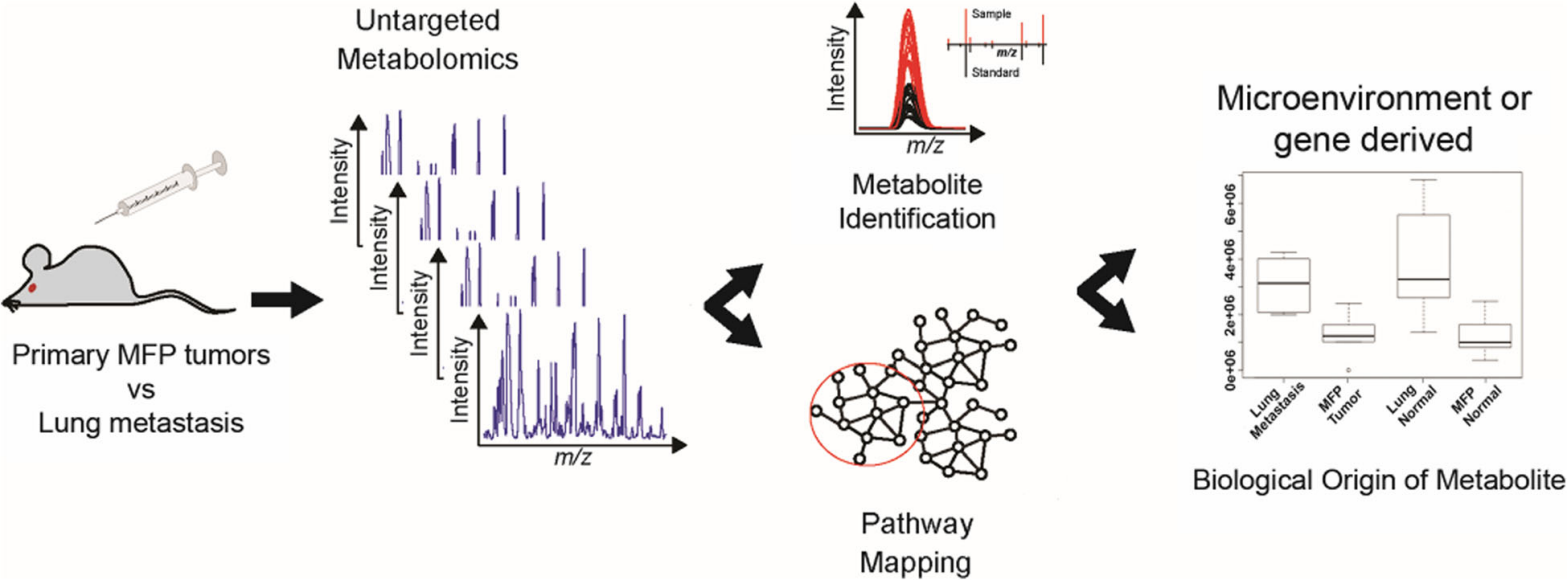
Overview of metabolomic workflow. Untargeted metabolomic analysis was carried out on tissue extracts from a mouse model of metastasis. Autonomous metabolomic and pathway analysis of paired tissues (primary tumor versus metastasis) revealed correlated metabolic pathway changes, in particular the increased production of cholesterol sulfate in metastasis

ESI source conditions were set as follows: gas temperature 200 °C, drying gas 11 l/min, nebulizer 15 psi, fragmentor 365 V, sheath gas temperature 300 °C, sheath gas flow 9 l/min, nozzle voltage 500 V, and capillary voltage − 2500 V. The instrument was set to acquire over the *m*/*z* range 60–1000 with a MS acquisition rate of 1.67 spectra/s. The data were processed using XCMS Online [12]. Paired parametric tests were performed, with a *p* value < 0.05 having statistical significance. The *q* value was also assessed to eliminate false positive results. Features were compiled in a feature list table and as an interactive cloud plot, containing integrated intensities (extracted ion chromatographic peak areas), observed fold changes across the two sample groups, and statistical significance for each sample. Tentative metabolic pathways were identified using mummichog version 0.10.3, available as part of the XCMS Online program. An autonomous metabolomic workflow was also carried out for each sample by HILIC-MS in ESI negative mode to obtain an automatic list of metabolite identifications post-run. These identifications are based on precursor mass and fragment ions [8]. Acquisition was as follows: MS mode acquisition rate 2.86 spectra/s; MS/MS mode acquisition rate 13.33 spectra/s at 20 eV with a narrow isolation width (1.3 Da); 10 max precursors per cycle, with active exclusion after 4 spectra, released after 0.15 min; and a threshold of 200 counts. Two contaminant ions with *m*/*z*’s 172.9297 and 248.9762 were excluded throughout the whole run. Metabolite identifications were further confirmed by comparing retention time and tandem MS to standard compounds. For targeted identification of selected precursors, the default isolation width was set as narrow (1.3 Da), with a MS acquisition rate at 2.87 spectra/s and MS/MS acquisition at 2.87 spectra/s. The collision energy was set to 20 and 40 eV on separate runs. An overview of the workflow can be seen in Fig. 1.

## Results

### Metabolomic analysis reveals increased lipid species in metastasis

**Fig. 2:**
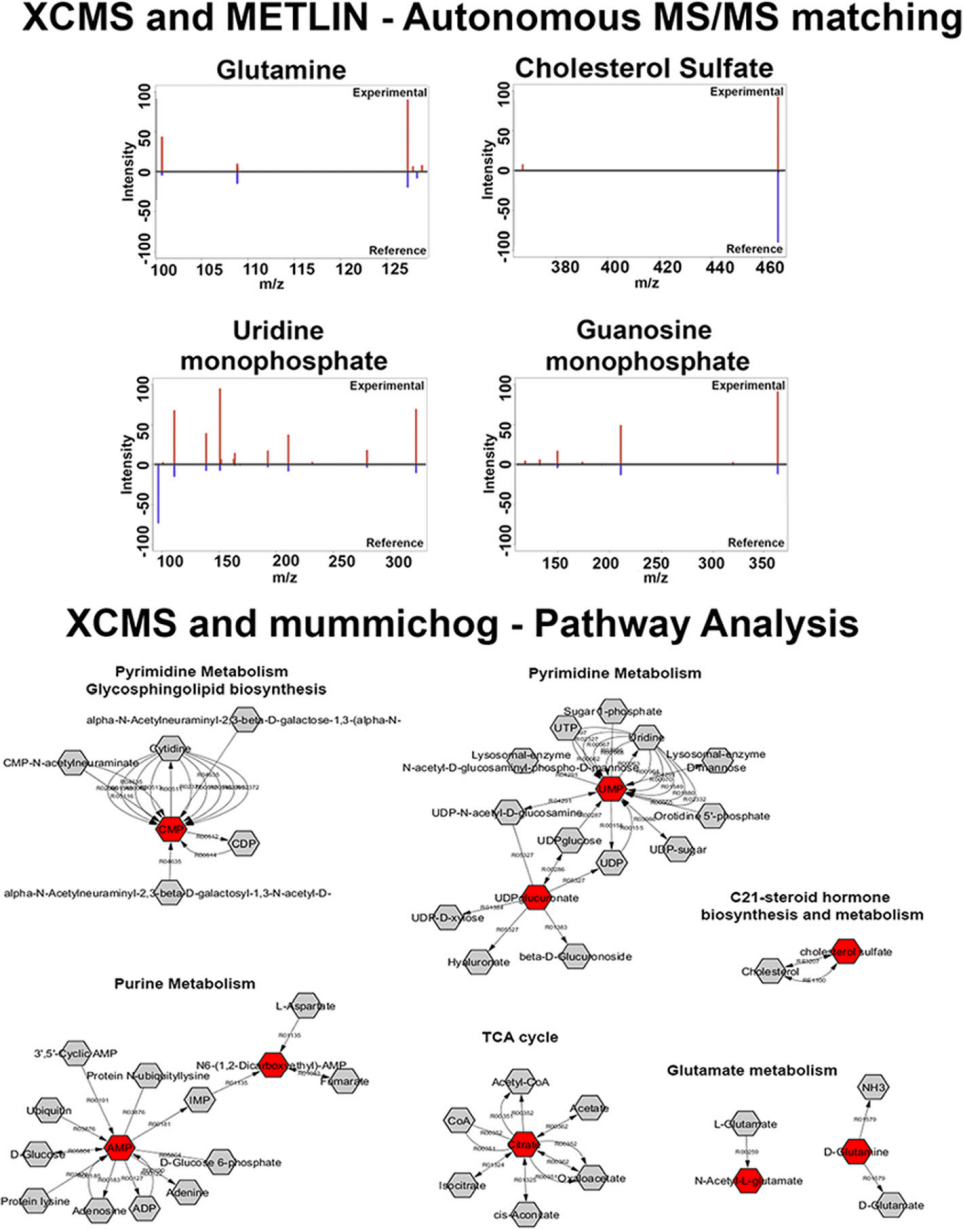
Paired untargeted metabolomics analysis of primary mammary fat pad tumors compared to lung metastasis (*n* = 4). Upper panel, autonomous metabolomics aids in the identification of metabolites by automated tandem MS matching to the METLIN database; the panels show experimental and reference tandem MS comparisons for glutamine, cholesterol sulfate, uridine monophosphate and guanosine monophosphate at a collision energy of 20 eV. Lower panel, mummichog pathway analysis integrated with XCMS Online software, reveals pathways that are putatively correlated to differences between primary and metastatic cancer cells

An untargeted metabolomic approach was used to identify metabolites in extracts of primary tumors and their spontaneous microdissected lung metastases (*n* = 4/group). Of note, animals were euthanized when there was a bioluminescence signal of 1 × 10^7^ photons/s/cm^2^ for the lung metastasis. Paired analyses revealed a total of 10,329 aligned metabolic features. Filtering to remove isotopes and noise prioritized 110 features that were significantly different in abundance between the two groups of samples (*p* value < 0.01, *q* value < 0.1, fold change > 2, intensity > 10,000, paired *t* test) (XCMS Online Job ID #1060717). In order to identify the features, an autonomous workflow was used which acquired both quantitative MS and qualitative MS/MS data. The data were simultaneously matched to MS/MS fragmentation patterns on the METLIN database [8]. Using this function, it was possible to identify uridine monophosphate (UMP), guanosine monophosphate (GMP), glutamine, and cholesterol sulfate (Fig. 2). In order to increase the identification of metabolite features, additional targeted MS/MS was carried out on precursor ions from the prioritized list, confirming the presence of citrate/isocitrate, N6-succino adenosine, cytidine monophosphate (CMP), 16:0 Lyso phosphoethanolamine (PE), and 16:0 phosphatidylglycerol (PG). These metabolites were further confirmed by comparison to authentic standards. Table 1 displays these metabolites, their significance, and *p* values in relation to their increased abundance in primary tumors or lung metastases. In order to expand on the analysis and provide biological meaning to the metabolites, we used the network mapping tool mummichog to assess interconnectivity of metabolites in biological pathways [13]. This software looks at patterns of related putatively identified metabolites (based on their *m*/*z* only) to lead further metabolite identification; it is integrated as part of the XCMS Online platform [12]. The top metabolite predictions made by mummichog were glutamine, CMP, GMP, UMP, citrate/isocitrate, and adenosine monophosphate (AMP). AMP was further confirmed against a standard. As the pathway analyses indicated an involvement of nucleotide (*p* = 0.00139), glutamate (*p* = 0.00336), and phosphocholine (*p* = 0.01843) metabolism, these pathways were investigated further and revealed significant differences to other metabolites housed in these pathways: uridine 5′-diphospho (UDP)-glucuronate, *N*-acetyl-l-glutamate, 16:0 Lyso PG (DDPG), and 16:0–18:1 PG (POPG) (Fig. 2). The LipidMaps database was used to further putatively identify DDPG, and POPG by MS/MS fragmentation patterns, and was confirmed by comparison to standards. Thus, the metabolites upregulated in metastases included phospholipids and cholesterol sulfate, and those upregulated in the primary tumor were involved primarily in glutamate metabolism and nucleotide synthesis. Additional file 1: Figure S1 shows the distributions for each of the metabolites.

**Table 1:** List of metabolites significantly changed in metastasis tissues (*n* = 4) compared to primary tumor tissues (*n* = 4), paired Welch’s *t* test

Metabolite name	Mass-to-charge ratio [M-H]^−^	Fold change	Retention time (min)	Direction of change in metastasis	*p* value
Cholesterol sulfate	465.3050	5.7	15.59	↑	4.00E−04
16:0 Lyso phosphatidylethanolamine	452.2771	3.0	16.67	↑	1.86E−03
DPPG (16:0 phosphatidylglycerol)	721.5012	15.2	17.11	↑	2.80E−03
POPG (16:0/18:1 phosphatidylglycerol)	747.5185	2.5	17.03	↑	1.41E−02
16:0 Lyso phosphatidylglycerol	483.2719	14.0	18.66	↑	1.68E−02
Cytidine monophosphate	322.0443	4.3	36.70	↓	9.20E−04
Citrate/isocitrate	191.0202	2.8	42.79	↓	1.20E−03
Guanosine monophosphate	362.0505	2.2	39.84	↓	1.24E−03
Uridine monophosphate	323.0286	3.8	36.96	↓	2.40E−03
N6-succinyl adenosine	382.0999	7.0	36.42	↓	4.44E−03
Glutamine	145.0621	1.8	21.63	↓	6.14E−03
*N*-acetyl-l-glutamate	188.0565	6.5	34.66	↓	4.32E−02
Adenosine monophosphate	346.0560	2.0	37.23	↓	4.92E−02
Uridine 5′-diphosphoglucuronate	579.0262	2.1	42.95	↓	4.99E−02

### The cancer cell microenvironment as a source of nutrients

**Fig. 3:**
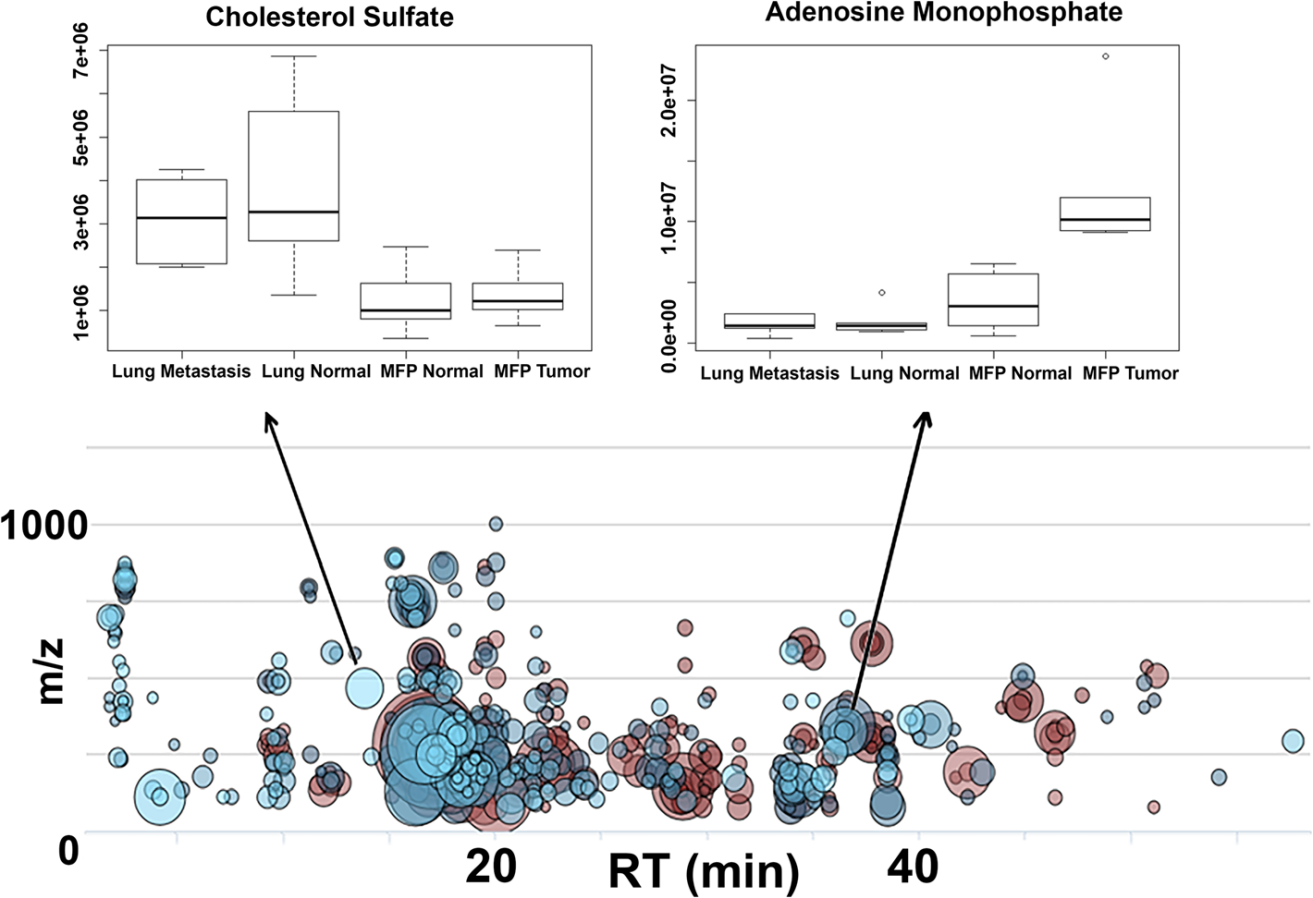
Multigroup analysis by XCMS Online. Cloud plot represents significantly altered metabolites, and box-and-whisker plots detail the relative abundance of metabolites in all four tissue types (ANOVA with Bonferroni correction, *n* = 5/group, *p* < 0.015)

**Fig. 4:**
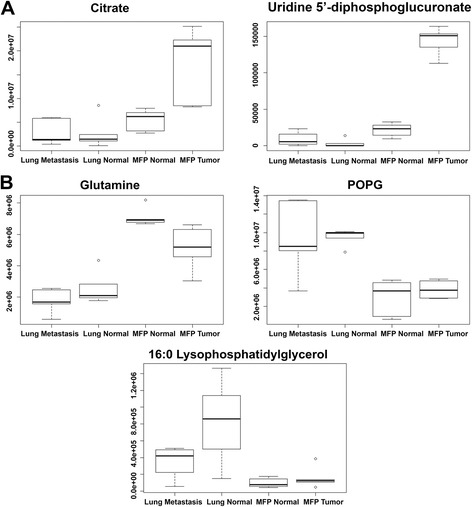
Distribution of relative abundances for metabolites in tumor and normal tissues. **a** Metabolites increased in primary tumor only. **b** Metabolites increased in normal stroma and tumor. Box-and-whisker plots generated by XCMS Online for a multigroup analysis (ANOVA, *n* = 5/group, whiskers, median with minimum-maximum; boxes, interquartile range) (POPG = 16:0/18:1 phosphatidylglycerol)

In order to assess the possibility of metabolite uptake from the microenvironment, samples were analyzed from normal MFP and lung tissues and compared to MFP tumors and lung metastasis (*n* = 5/group). The XCMS Online platform was used to perform multigroup analysis by ANOVA (Job ID: 1095497) revealing the distributions of the metabolites in the tissues [14]. Figure 3 shows a cloud plot of all the statistically significant filtered features. It can be seen that a number of metabolites that were increased in the metastasis or primary tumors were at similar abundances in their normal surrounding tissues. These metabolites include glutamine, cholesterol sulfate, and phospholipid compounds, indicating that the microenvironment could be a source of metabolites for cancer cells (Figs. 3 and 4). There were also a number of metabolites that were only significantly upregulated in tumor tissues and not in normal tissues: citrate/isocitrate, UDP-glucuronate, and mononucleotides Figs. 3 and 4).

## Discussion

The metabolism of a cancer cell depends on both its genetic reprogramming during malignant transformation and tumor progression, as well as on its interactions with the tumor microenvironment. In this study, we used an autonomous metabolomic workflow to uncover the metabolic differences between cancer cells in a primary tumor, and those that have metastasized to a distant organ. Paired analysis of primary MFP tumors and lung metastases in a xenograft model revealed a number of significantly altered metabolites between the tissues. Notably, cholesterol sulfate was highly upregulated in spontaneous pulmonary metastases compared to their paired primary tumors and thus raised interest for further investigation due to its previously implicated roles in cancer invasion and cell signaling. Cholesterol sulfate is a metabolic product of cholesterol and, along with phospholipids and ceramides, is integral to maintaining the cellular plasma membrane and determines its elasticity. This metabolite has known signaling functions and can modify the activity of serine proteinases of the coagulation cascade [15, 16]. Thus, it can potentially promote platelet interaction with circulating cells including disseminating tumor cells within the bloodstream [15, 16]. Cholesterol sulfate can also accelerate the proteolytic activity of matrix metalloproteinase-7 towards selective substrates in the extracellular matrix, thus potentially aiding in metastasis [17], and can regulate protein kinase C, which is involved in cellular differentiation and carcinogenesis [18].

In our study, comparison of normal and tumor tissues also revealed that cholesterol sulfate was higher in both normal lung tissue extracts and lung metastases compared to MFP. The lung metastases were microdissected and validated to contain only minimal traces of normal lung tissue. Therefore, the presence of this metabolite in pulmonary lesions could have arisen by uptake from the extracellular environment as well as by cancer cell production. For the latter hypothesis, it is possible that a subset of cells within the primary tumor have increased expression of cholesterol sulfotransferase, facilitating the production of cholesterol sulfate and thereby supporting hematogenous dissemination of metastatic cancer cells to the lung.

In addition to the roles of cholesterol sulfate itself, the enzyme sulfotransferase (SULT)2B1b which has a high affinity for cholesterol sulfonation has also been linked to carcinogenesis [19]. Recent studies in mice have shown that prostate cancer cells with RNAi-mediated knockdown of SULT2B1b decrease cell growth and induce cell death [20]. In addition, SULT2B1b promoted proliferation of hepatocellular carcinoma cells [21], decreased migration of a metastatic non-small cell lung cancer cell line [22], and promoted angiogenesis in gastric cancer [23] and cell growth and invasion in colorectal cancer [24]. Studies also show that patients whose tumors express higher levels of SULT2B1b have a worse prognosis [22–25]. There is strong evidence that indicates a role for both SULT2B1b and cholesterol sulfate in cancer; our hypothesis is that the target organ uses cholesterol sulfate to facilitate seeding of metastatic cancer cells.

In addition to changes in cholesterol sulfate, phospholipids were also highly upregulated in metastases. They were also similarly elevated in normal lung tissues and lung metastases. Phospholipids have been previously associated with cancer, with proposed mechanisms in protein trafficking, promoting the onset and progression of the disease [26]. They have also been associated with metastatic cancer cells [27]. It has been previously demonstrated that fatty acids are synthesized de novo for phospholipid and subsequent cell membrane synthesis. However, a recent study showed that proliferating cells scavenge highly abundant fatty acids from the extracellular environment for phospholipid synthesis, in contrast to cells that are surrounded by low concentrations of fatty acids and carry out de novo synthesis [28]. Conversely, cancer cells can scavenge unsaturated fatty acids from lysophospholipids [29]. In addition, studies have shown that cancer cells use macropinocytosis to internalize extracellular proteins which then undergo proteolytic degradation, yielding amino acids for growth [30, 31]. Thus, it is possible that it is more efficient for cancer cells to take up nutrients from the microenvironment, where they are in high supply, rather than, or in addition to, utilizing intracellular resources for de novo synthesis [32]. Likewise, in normal MFP tissues, glutamine was at similar abundance levels to those found in the primary MFP tumors, indicating that these cancer cells may also use the microenvironment as a source of energy-rich metabolites. However, there were a large number of metabolites that were only increased in the primary tumors and not present in normal tissue:citrate/isocitrate, AMP, GMP, CMP, UMP, and UDP-glucuronate indicating that these metabolites are generated intracellularly by the tumor cells and are either not taken up from the microenvironment or are potentially products of other metabolites.

As aforementioned, there were several metabolites involved in DNA/RNA synthesis and the TCA cycle that were elevated in primary tumors compared to metastases. This indicates that the metastases have depleted nutrients available for nucleotide synthesis, including essential cofactors such as NADPH. De novo lipogenesis, which similarly requires high levels of NADPH, is shut down to preserve NADPH for nucleotide synthesis and cancer cell survival; therefore, cells use alternative methods to scavenge nutrients for growth, such as fatty acids from phospholipids [29, 33]. Fatty acids derived from phospholipids can supply more energy to the cell through mitochondrial oxidation than from oxidation of glucose or amino acids, so are an important nutrient source for the tumor cell [34]. This hypothesis is further supported by the decreased levels of both glutamine and citrate by the metastases; glutamine can undergo reductive carboxylation to citrate for lipid generation [35]. Phospholipid scavenging has previously been reported for cells that are under hypoxic stress and require additional nutrients for growth [29]. Cells that have constitutive mTORC1 signaling and hypoxia also have been shown to have a dependency on extracellular desaturated fatty acids to support protein synthesis [36]. Of importance, tumor cells that acquire lipids through extracellular scavenging are resistant to stearoyl-CoA desaturase 1 (SCD1) inhibitors [29].

The metabolic changes that occur in cancer cells that gain metastatic activity have been reported in the literature, and it is evident that tumor cell metabolic rewiring is dependent on the organ in which the metastatic cells are seeded. A recent study showed that breast cancer cells which metastasize to the liver favor glycolysis, while those that metastasized to the lung and bone favor oxidative phosphorylation (OXPHOS) [37]. This difference is likely due to genetic features that underlie specific cancer subtypes. A study of clinical samples from 15 cancer types in the Cancer Gene Atlas consortium showed that suppression of OXPHOS genes was a common feature of metastases when comparing to primary tumors and correlated with poorer clinical outcome [38]. It also confirmed that metastasis is not only dependent on genetics but also on the metabolic environment of the new organ site.

## Conclusions

Metabolic reprogramming, recognized as a typical hallmark of cancer cell metabolism, is influenced by an interplay between tumor microenvironment and genetic complement that has given rise to the developments of a new generation of therapeutics. Herein, this study reports a model for rapid identification of metabolites in primary tumors and metastases and has shown that cholesterol sulfate and phospholipid species are highly upregulated in metastasis. Our hypothesis is that tumor cells scavenge fatty acids, as an alternative energy source concomitant with observed decrease of nucleotides, glutamine, and citrate, indicating a nutrient-depleted metastatic phenotype associated with tumor aggression. Future studies will further assess the role of cholesterol sulfate and phospholipids in fueling metastatic cancer and the involvement of transporters in mediating the flow of metabolites between disseminating tumor cells and their microenvironments.

## Additional file

Additional file 1: Figure S1. Distributions of relative abundance for each confirmed metabolite. Box-and-whisker plots were generated by XCMS Online (whiskers, median with minimum-maximum; boxes, interquartile range). (PG phosphatidylglycerol, PE phosphoethanolamine, DPPG 16:0 phosphatidylglycerol, POPG 16:0/18:1 phosphatidylglycerol.) (TIFF 357 kb)

## Abbreviations

HILIC: Hydrophilic interaction liquid chromatography
MFP: Mammary fat pad
SULT: Sulfotransferase
