# Supplementary figures and images for: Metabolomics guided pathway analysis reveals link between cancer metastasis, cholesterol sulfate, and phospholipids
Source: Cancer Metab. 2017 Oct 31;5:9. doi: 10.1186/s40170-017-0171-2 (PMC5663111; doi:10.1186/s40170-017-0171-2)

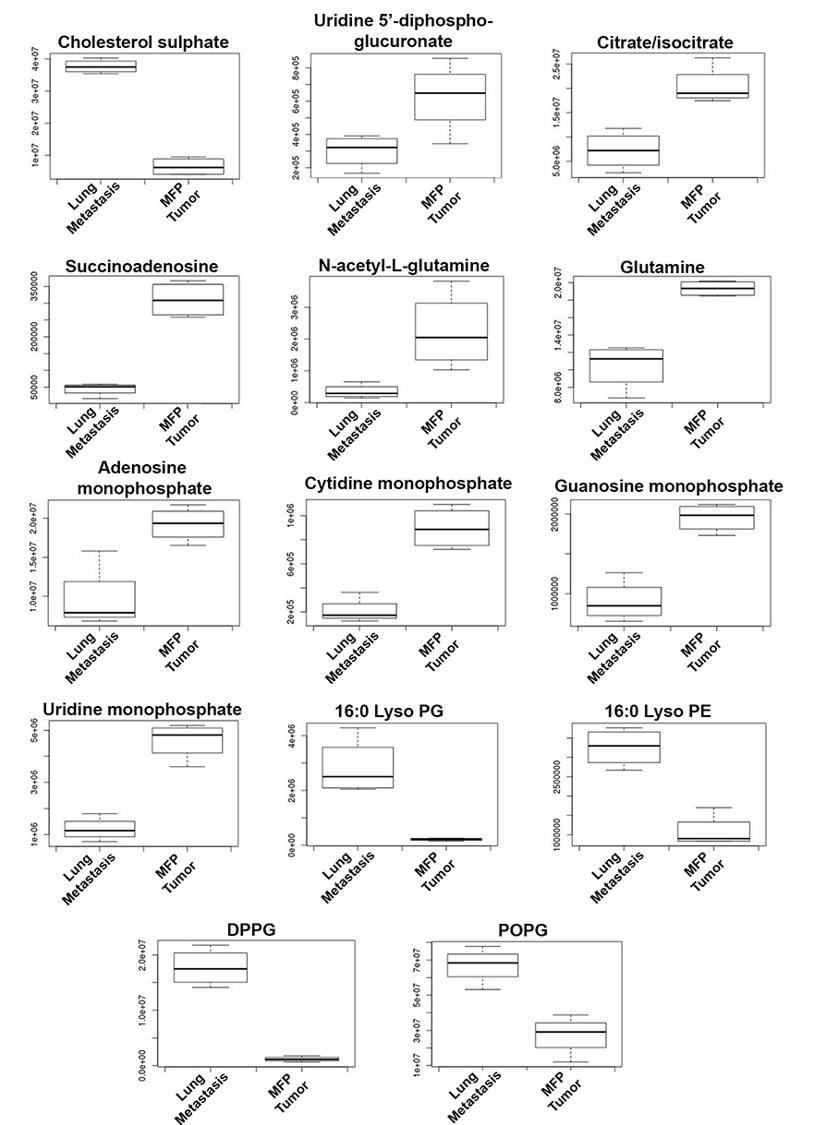

Supplement: Additional file 1: Figure S1. — Distributions of relative abundance for each confirmed metabolite. Box-and-whisker plots were generated by XCMS Online (whiskers, median with minimum-maximum; boxes, interquartile range). (PG phosphatidylglycerol, PE phosphoethanolamine, DPPG 16:0 phosphatidylglycerol, POPG 16:0/18:1 phosphatidylglycerol.) (TIFF 357 kb) [file 40170_2017_171_MOESM1_ESM.tif]
